# Psychological stress associated with prognostic uncertainties in recently diagnosed Parkinson’s disease patients: A qualitative study

**DOI:** 10.1371/journal.pone.0319576

**Published:** 2025-03-11

**Authors:** Yasmin H. Aboelzahab, Azadeh Bojmehrani, Yomna Elsheikh Ahmed, Heather Boon, Connie Marras, Richard Foty

**Affiliations:** 1 Department of Laboratory Medicine and Pathobiology, Temerty Faculty of Medicine, Translational Research Program, University of Toronto, Toronto, Ontario, Canada; 2 Leslie Dan Faculty of Pharmacy, University of Toronto, Toronto, Ontario, Canada; 3 Division of Neurology, Department of Medicine, University of Toronto, Toronto, Ontario, Canada; 4 Toronto Western Hospital, University Health Network, Toronto, Ontario, Canada; BSMHFT National Centre for Mental Health, UNITED KINGDOM OF GREAT BRITAIN AND NORTHERN IRELAND

## Abstract

**Background:**

Parkinson’s disease (PD) is a common neurodegenerative disorder that negatively impacts thousands of patients in Canada. The unexpected nature of PD is associated with a decline in mental health. The highest level of psychological stress occurs during the early years following the diagnosis.

**Objectives:**

To understand the psychological stress associated with prognostic uncertainties in recently diagnosed PD patients, uncover the gaps in the current support systems, and recommend areas for improvement in the support services that aim to decrease the psychological stress associated with receiving the PD diagnosis.

**Methods:**

An exploratory qualitative study was conducted using semi-structured interviews with 13 PD patients diagnosed for more than 6 months and less than 5 years. Participants were recruited from the Toronto Western Hospital Movement Disorders Clinic, Toronto, Ontario, Canada until saturation of key themes was reached.

**Results:**

Five major themes were identified capturing the lived experiences of PD patients following diagnosis: 1) the circumstances of receiving the diagnosis and its psychological impact on PD patients, 2) the impact of intrapersonal factors on the PD journey, 3) the role of social relationships in PD patient’s life, 4) the interaction of PD patients with different elements of the healthcare system, and 5) support services available for recently diagnosed PD patients.

**Conclusions:**

This study uncovers the psychological burden faced by PD patients due to prognostic uncertainties and insufficient support systems. It emphasizes the importance of a patient-centered approach for improving their quality of life and healthcare experiences through personalized support services.

## Introduction

Parkinson’s Disease (PD) is a progressive neurodegenerative disorder that has a major impact on quality of life [1,2]. PD is primarily characterized by a range of motor symptoms, which are categorized into primary and secondary [3,4]. Primary motor symptoms include akinesia, bradykinesia, tremor, rigidity, and postural instability [4]. Secondary symptoms such as gait disturbances, micrographia, impaired precision grip, and speech difficulties, further contribute to the physical challenges faced by PD patients [4]. These motor symptoms are caused by the progressive loss of dopaminergic neurons in the substantia nigra, a critical region of the brain responsible for motor control [3,4]. Over time, these symptoms impair mobility, balance, and coordination, further contributing to the challenges of daily life for patients [5].

In addition to motor symptoms, PD patients frequently experience cognitive impairments, including difficulties with executive functions, memory, and attention [6,7]. Cognitive decline can vary in severity, ranging from mild cognitive impairment to dementia in advanced stages of the disease [8]. These non-motor manifestations impact patients’ independence and quality of life, often exacerbating the psychological burden associated with the condition [6].

Psychological difficulties are a critical and often overlooked aspect of PD [9]. Common psychological issues include depression, anxiety, apathy, and, in some cases, impulse control disorders which can affect patients’ mental health and overall quality of life [9,10]. These difficulties can occur at any stage of the disease, sometimes even preceding a formal diagnosis by several years, and they can be just as disabling as the motor symptoms [9].

In addition to these well-known challenges, subtler factors also contribute to psychological distress in PD patients, both at the time of diagnosis and throughout the disease course. Difficulties with nutritional management such as maintaining adequate oral intake and adapting to dietary needs, can exacerbate distress and impact overall health [11]. Similarly, medication adherence in PD patients is closely tied to their perceived control over the disease [12]. Those who feel they have a sense of agency in managing their symptoms and treatment plans are more likely to adhere to their prescribed medications [12]. Conversely, patients who view their condition as uncontrollable may face challenges with adherence, which can exacerbate psychological stress and negatively impact their overall well-being [12].

Moreover, societal emergencies such as the COVID-19 pandemic, have amplified illness uncertainty and psychological distress among PD patients [13,14]. Disruptions to healthcare access and social support systems heightened concerns around hospitalization, loss of independence, and timely access to medications [13]. These fears were often driven by prior negative experiences or anxiety about competing healthcare priorities during the lockdown period [13].

PD symptoms typically tend to worsen over time, but the disease progression varies between individuals, creating a substantial level of uncertainty [2,15]. Disease uncertainties raise questions about what the future may hold for a Parkinson’s patient with regards to diagnosis, progression, prognosis, treatment outcome, and how the disease may adversely affect their life. The unpredictable nature of the disease can exacerbate mental health challenges and patients may become apprehensive and anxious about their future [16,17]. As reported by Parkinson’s UK, the highest level of psychological stress occurs during the early years following diagnosis, especially within the first two years [18]. The anxiety and stress stemming from this disease’s uncertainties tend to decline over time, making recently diagnosed patients more susceptible to experiencing heightened psychological stress [18].

An environmental scan was conducted as a needs assessment tool to explore the psychological stressors associated with receiving the diagnosis of PD and identify the support services available for recently diagnosed PD patients [19]. This involved exploratory discussions with key stakeholders including PD patients, caretakers, and representatives from Parkinson’s Canada and Parkinson’s UK. These discussions were not part of the formal methodology but served to verify the significance of the problem and validate the need to investigate psychological stressors as a real and pressing issue. Three primary sources of psychological stress were identified: diagnostic uncertainties of the disease, prognostic uncertainties, and treatment outcomes (data not published). This preliminary work ensured the study would address a meaningful gap and provided foundational insights to shape the study’s design and focus. Moreover, it helped refine the research questions and clarified the scope of the investigation.

This study is focused on prognostic uncertainties as the main problem to explore. Patients experience prognostic uncertainties due to the cumulative challenges associated with the disease progression whether personal, professional, or social difficulties. Such uncertainties contribute to heightened psychological stress during the early years following diagnosis [20]. The diagnostic period is crucial as it determines the course of treatment and its effectiveness and affects the patient’s sense of identity, relationships, and understanding of their future [21]. PD’s progression is inherently unpredictable, with variability in symptoms and outcomes across individuals, making it challenging for patients to anticipate what lies ahead [20–22]. This uncertainty often fosters a fear of the unknown, as patients worry about how the disease will impact their abilities, independence, and quality of life, including concerns about losing autonomy, struggling in their profession, or becoming a burden to their caregivers or society [13,18,20]. Comparisons with other PD patients can exacerbate this anxiety, leading to catastrophizing and an increased risk of depression and demoralization [22]. Furthermore, while some predictors of disease progression exist such as motor and cognitive symptoms, relying on these may unintentionally increase stress by emphasizing negative outcomes [22].

Despite increasing awareness of the psychological impacts of PD, existing research predominantly focuses on motor symptoms and treatment efficacy. Studies examining the role of prognostic uncertainty in psychological stress are limited, and the early diagnostic period a critical window of vulnerability remains underexplored [15,16]. This study aimed to understand the psychological stress associated with prognostic uncertainties in recently diagnosed PD patients, uncover the gaps in the current support systems, and recommend areas for improvement in the support services that aim to decrease the psychological stress associated with receiving the PD diagnosis.

## Materials and methods

### Study design

We conducted an exploratory qualitative study to investigate the psychological stressors associated with prognostic uncertainties of newly diagnosed PD patients [23]. The patient’s perspective is highly important in uncovering various aspects of psychological stress associated with a PD diagnosis. We therefore chose to conduct in-depth interviews with recently diagnosed PD patients to explore each patient’s unique experience and allow them to share their thoughts in a private setting [24,25]. In-depth interviews are designed to explore comprehensive perspectives, experiences, and motivations through detailed, open-ended interactions between researchers and participants. This method focuses on achieving a deep understanding by following a semi-structured interview guide with probing questions that encourage participants to share their thoughts and feelings thoroughly [26,27].

## Theoretical framework

This study retrospectively applied the Uncertainty in Illness Theory (UIT) to interpret the psychological stress experienced by recently diagnosed PD patients [28]. UIT stated that uncertainties arise when individuals struggle to assign meaning to illness-related events due to insufficient, inconsistent, or unpredictable information [28,29]. UIT also provided a structured lens to explore the psychological burden of prognostic uncertainties and the adapting strategies by focusing on how individuals evaluate situations, use coping mechanisms, and experience outcomes. Applying this theory retrospectively enabled a deeper understanding of how uncertainty shaped participants’ experiences and stress levels.

### Sampling strategy

Our target population for the study was recently diagnosed PD patients. To ensure the richness of data and capture diverse perspectives, we continued data collection until thematic saturation was reached. In this context, “saturation” was defined as the point at which a pattern of repetition starts to emerge in the data, and no new themes directly related to our research question/aims [30,31]. Saturation was assessed through ongoing analysis during the data collection process, allowing the researchers to recognize repetitive patterns in participants’ responses and determine that additional interviews were unlikely to yield novel insights [32].

Additionally, we employed the concept of ‘information power’ to guide the adequacy of our sample size. This approach suggests that the more relevant information the sample holds for the study, the fewer participants are required [33]. Given the focused aim of our study, the specificity of the sample (recently diagnosed PD patients), and the rich data obtained through semi-structured interviews, we initially set a target sample size of 10–15 participants, The sample size was finalized at 13 PD patients after thematic saturation was reached.

Purposeful sampling was used to select participants most likely to yield relevant and rich data [34,35]. The sampling criteria were defined to ensure participants met specific eligibility requirements related to their recent diagnosis and ability to contribute meaningful insights into the research question. Inclusion criteria required participants to have received their PD diagnosis within more than 6 months and less than 5 years from the start of the study. This range allowed for a degree of heterogeneity in the sample, capturing diverse experiences and perspectives depending on the time elapsed since diagnosis. Participants needed to have been diagnosed with PD for at least 6 months to ensure they had sufficient time to process their diagnosis. This period allowed participants to reflect on their initial reactions, adapt to their diagnosis, and engage with available support systems, enabling them to provide richer and more meaningful insights into their experiences.

Participants were formally registered patients at the Toronto Western Hospital (TWH) Movement Disorders Clinic, spoke English, lived in Ontario, Canada, were willing to provide informed consent, and could participate through internet-based platforms. Exclusion criteria ensured the cognitive and neurological health of participants by excluding individuals with cognitive impairments, as judged by their treating physician, or those with other active neurological diseases. While the sample was homogenous in terms of its focus on recently diagnosed PD patients, it was heterogeneous in other aspects such as age (ranging from 52 to 75 years), gender, and the time since diagnosis. Eligibility was determined through a review of clinic records and input from treating physicians, followed by direct outreach to potential participants.

### Recruitment

PD patients were recruited from the TWH Movement Disorders clinic. The clinic research coordinators were informed of the study’s aim and inclusion criteria to assist in selecting eligible participants. The clinic routinely offered patients the opportunity to consent to a review of their medical records for research purposes and to be contacted if they qualified for a study. Patients who met the inclusion criteria and had provided consent to be contacted for research were approached by a member of the research team via email or telephone. The study’s purpose and nature were explained to them and detailed information was provided to those who expressed interest in participating.

To reduce the potential of any possible power dynamics, patients were informed that their physicians were not involved in conducting the study, and their data was to be anonymized once the data analysis process was complete. Participants did not previously know the researchers conducting the study. The recruitment information included a summary of the study and its main goal as well as names and roles of the study researchers who were conducting the interviews. Recruitment started on June 22, 2022, and ended on August 15, 2022.

### Data collection

Semi-structured interviews were conducted to understand the experiences of participants living with PD [25,36]. The interview questions were designed to understand the psychological stress associated with PD diagnosis. In addition to collecting participants’ demographics (Table 1), open-ended questions explored the main stressors of this stage and the role of prognostic uncertainties in raising the psychological stress level among recently diagnosed PD patients [37]. Questions were also asked about the support services they received at the time of their diagnosis and their effectiveness. Participants were asked for any suggestions they may have on how to improve the current support services provided for recently diagnosed PD patients as a final question to each interview. No interview was repeated and each interview lasted 30 to 40 minutes, aligning with the typical duration for in-depth interviews [38].

**Table 1:** Demographic information of participants.

Participant	Gender	Age (Years)	Diagnosis Year	Pseudonym
01	Female	54	2018	FP1(54yr)
02	Male	69	2019	MP2(69yr)
03	Female	65	2017	FP3(65yr)
04	Female	56	2019	FP4(56yr)
05	Male	75	2017	MP5(75yr)
06	Female	70	2020	FP6(70yr)
07	Female	69	2022	FP7(69yr)
08	Male	71	2020	MP8(71yr)
09	Male	70	2019	MP9(70yr)
10	Male	55	2019	MP10(55yr)
11	Male	73	2017	MP11(73yr)
12	Male	52	2020	MP12(52yr)
13	Male	67	2019	MP13(67yr)

A prepared interview guide was followed including main open-ended questions that focused on the study topic and follow-up questions designed to elicit more detailed responses and encourage participants to elaborate on their experiences. These questions facilitated comparison across participants and minimized pivoting from the study focus. Not all questions in the guide were asked during every interview; this flexibility allowed the flow to adapt to each participant’s responses, enabling them to take their time and provide in-depth answers.

The main questions aimed to understand the psychological stress associated with a PD diagnosis, explore the primary stressors at this stage, and examine the role of prognostic uncertainties in elevating stress levels. Participants were also asked about the support services they received at the time of diagnosis, their perceived effectiveness, and suggestions for improving these services. The interview guide was pilot-tested through a mock interview with a research team member who is also a caregiver for a PD patient but was not involved in the initial development of the questions. Feedback from this pilot test assessed the clarity, relevance, and flow of the questions, as well as the effectiveness of probing techniques. The semi-structured nature of the guide allowed for ongoing refinement and updating throughout the interview stage to address any ambiguities or gaps identified during the interviewing process (S1 Appendix).

All interviews were conducted virtually using Microsoft Teams, with two of the three researchers (YHA, AB, YEA) conducting each interview in alternating pairs (e.g., YHA and AB for one interview, AB and YEA for another, and YEA and YHA for another). The primary interviewer was responsible for asking the main questions listed in the interview guide. The secondary interviewer asked probing questions that the primary interviewer might have missed. In doing so, we were able to exchange feedback and reflect on our interpretations of the participants’ answers after each interview. Only the participants and the interviewers attended the interviews. Interviews were conducted between June 29 and August 17, 2022.

### Data analysis and reporting

Data analysis was conducted in parallel with the interviews to continually review and adjust the interview guide. Field notes were also recorded after each interview to capture additional observations, contextual details, and initial impressions that might not have been explicitly mentioned by the participants. All interviews were video-recorded and transcribed via Microsoft Teams. No transcripts were returned to participants for comment and/or correction.

Conventional content analysis was used to analyze the qualitative study data [39]. This approach is particularly well-suited for exploring phenomena with limited existing literature or prior research such as the psychological stress associated with prognostic uncertainties in recently diagnosed PD patients [39]. By employing inductive reasoning, this method avoids the imposition of predefined coding systems for interpreting the data [39].The data analysis process began with an iterative reading of the interview transcripts to achieve comprehensive immersion and assimilation of the entire dataset [39]. Only the research team members involved in the analysis process (YHA, AB, YEA) had access to the interview transcripts. Initial codes were then inductively generated directly from the data to capture the main themes and relevant sub-themes. These codes were regularly updated to incorporate any newly identified ideas or patterns [39].

**Fig 1:**
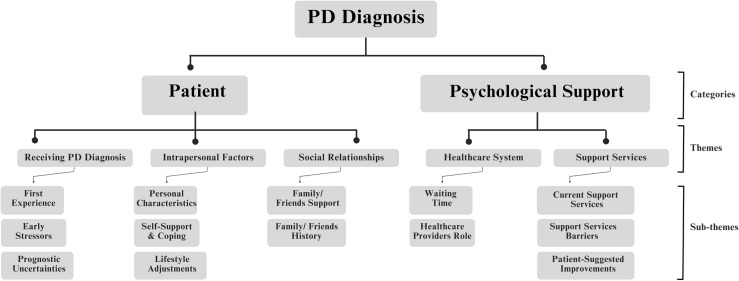
Hierarchical coding tree of categories, themes, and sub-themes.

The coding process involved several steps to ensure reliability and consistency. Initial codes were generated by YHA and then reviewed and refined collaboratively with AB and YEA to establish agreement on definitions and applications. A subset of transcripts was independently coded by two of the researchers involved in the analysis process (YHA, AB, YEA). Coding outputs were compared between the researchers and any discrepancies were resolved through discussion until a consensus was reached. The final coding framework was developed which included the codes along with their definitions and descriptions (S2 Appendix). The identified themes and sub-themes were then examined and organized into a hierarchical structure under relevant categories (Fig 1).

This study has followed guidance offered by the Consolidated Criteria for Reporting Qualitative Research (COREQ) checklist (S3 Appendix) for reporting and interpreting the study findings [40]. A summary of the study results was shared with the participants; however, no feedback was collected from them.

Following an approach adapted from Morton et al., participants were provided with pseudonyms to ensure anonymity while conveying relevant demographic information [41]. Each pseudonym includes the participant’s gender (Male [M] or Female [F]), a participant number (e.g., P1), and age (e.g., 54yr). “FP1 (54yr)” represents a 54-year-old female participant (Table 1).

## Reflexivity

Our team was composed entirely of female researchers with diverse backgrounds in pharmacy, pediatrics, and bioengineering, and personal experience in PD caregiving. Three members (YHA: BScPhm, AB: PhD, YEA: MB BCh BAO) were master’s students with relatively new exposure to qualitative research (1–2 years of experience), though AB had prior experience in PD care research. Two team members had medical backgrounds, which facilitated understanding medical details such as medications and healthcare processes, enabling a more nuanced exploration of participants’ experiences.

This range of expertise was crucial in designing the study, recruiting participants, and collecting data. YHA, AB, and YEA conducted the interviews and data analysis. Our collective experiences, particularly the caregiving background of one team member, brought heightened empathy and a deeper understanding of participants’ narratives. However, these personal and professional experiences may have introduced potential biases such as assumptions about the challenges faced by PD patients or a tendency to overemphasize emotional stressors. To mitigate these biases, we engaged in regular team discussions to critically examine our interpretations and ensure they were grounded in the data. Additionally, having diverse perspectives within the team helped balance potential biases by allowing for critical peer review and alternative viewpoints during the analysis.

No prior relationship was established with participants before the study commenced. Participants were informed that the purpose of the study was to help establish a foundational block to support recently diagnosed PD patients and decrease the psychological stress associated with receiving the PD diagnosis. The ontological position of our team was rooted in constructivism which views reality as a social construct influenced by individual experiences and interactions [42,43]. Epistemologically, the team adopted an interpretivist approach, emphasizing the understanding of participants’ lived experiences and meanings attributed to their psychological stress and prognostic uncertainties [42,43]. To enhance the study’s trustworthiness, the components were critically reviewed by the study team supervisors, which included a qualitative expert, a neurologist, and a translational research expert.

## Ethical considerations

Informed written consent was obtained from all participants. REB approval was granted from both the University Health Network (REB# 22-5105) and the University of Toronto (REB# 35399).

## Results

### Study sample

Thirteen participants (8 men and 5 women) aged between 52 to 75 years old participated in the study. The length of time elapsed since PD diagnosis ranged from 6 months to 5 years at study time (Table 1). We interviewed all eligible participants via MS Teams. Data saturation was achieved after interviewing 13 participants, leading us to conclude the interview process. None of the participants refused to answer any questions and none withdrew from the interview or the study.

### Identified themes and sub-themes

Upon analysis of the interview transcripts, we initially generated 15 codes that captured the main ideas and patterns in the data. These codes were subsequently refined and organized into 5 key themes and 13 sub-themes. The themes were grouped into two main categories: “Patients” which included three themes, and “Support Services” which included two themes. This systematic coding process ensured that the themes accurately reflected the data. The hierarchical coding tree illustrating the categories, themes, and sub-themes is shown in Fig 1.

#### Circumstances of receiving the diagnosis and its psychological impact on PD patients.

One of the major identified themes was about the circumstances, timing, and questions participants had when receiving their diagnosis and how these impacted their PD journey. This theme included three sub-themes: first experience, early stressors, and prognostic uncertainties.

***First experience:*** The initial experiences of receiving a PD diagnosis marked the beginning of a life-changing journey for many participants. Although varied in their extent, participants’ initial feelings after receiving the PD diagnosis were mostly sadness, shock, and denial, reflecting the profound psychological burden of the news.

The touching words of FP1(54yr) captured this:

*“When I was diagnosed with Parkinson’s disease, I was terrified, I was horrified, I was destroyed.”*

MP2(69yr) talked about being in a state of Denial:

*“When I did get the first diagnosis from the neurologist. I sort of didn’t believe it.”*

For some, the diagnosis immediately led to existential concerns, FP4(56yr) also explained:

“I started immediately kind of thinking about my death. To be honest, that’s what I started to think about. How much time do I have left? What do I need to do? What do I need to do to make this OK for my family?”

***Early stressors:*** We identified a range of early stressors that compounded the emotional disturbance following their diagnosis. Participants attributed the main source of stress to their initial lack of knowledge about PD, MP13(67yr) shared:

*“I think I was just shocked. I didn’t know anything about Parkinson’s. I don’t know anybody that has it. It was just kind of a little bit of that strange unknown feeling. I started Googling and researching what does this even mean?”*

Several participants expressed their fear of becoming a burden on their family as the biggest stressor. FP3(65yr) even considered medically assisted death:

*“I wanted medically assisted death when I first got the diagnosis because I wanted to make sure that I’m not a liability to my family.”*

***Prognostic uncertainties:*** Receiving a PD diagnosis initiated a state of uncertainty related to disease prognosis. In this respect, participants listed personal, professional, and/or social uncertainties that accompanied PD progression. These unanswered questions were one of the main reasons behind the elevated stress levels in recently diagnosed PD patients. FP1(54yr) stated:

*“I don’t know. It’s just the what-ifs. What if tomorrow? What if? What if I lose my function? What if I can’t do my hobbies? What if I can’t feed my family? What if it was all of that? And it still is. You know it, it really still is, but at that. At that time, that was my biggest stress.”*

MP13(67yr) also highlighted the challenges of unpredictable outcomes:

*“The uncertainty of not knowing and nobody telling you definitively what to expect. Everybody experiences it differently. You might have stiffness, you might not, you might have speech problems, you might not. You might have hallucinations from your medications, or you might have nausea. There are so many possibilities and nothing to help you plan. You can’t plan your future, you can’t plan how you might react to medications, how your symptoms might progress.”*

#### The impact of intrapersonal factors on the PD journey.

The response of participants to PD diagnosis and the subsequent journey was mainly affected by their intrapersonal factors. This theme was focused on how each participant lived their own PD experience, which was influenced by a variety of patient-specific factors including educational background, profession, health literacy, daily routine, life goals, and coping strategies. Three sub-themes were identified: personal characteristics, self-support and coping, and lifestyle adjustments.

***Personal characteristics:*** Participants’ educational and professional backgrounds played a key role in shaping their initial responses to their diagnosis. Understanding these personal characteristics provided valuable contextual insights into the determinants of psychological stress levels among participants. For example, MP11(73yr) who had a medical background had misdiagnosed himself, causing the formal PD diagnosis to be delayed, he shared:

*“I thought I understood my symptoms well enough, but I kept thinking they were due to other conditions. It ended up taking me way too long to get the right diagnosis.”*

FP4(56yr) stated that her profession was a barrier to seeking help, she shared:

*“I’m a lawyer, so it’s not really easy for me to reach out to ask for help, right?”*

***Self-support and coping:*** Many participants independently educate themselves about PD and found support services including educational resources on the internet where they found some answers to their questions and concerns. MP5(75yr) described his extensive efforts to learn about the disease, he said:

*“I did a deep dive into the literature. I visited social media sites, the Michael J. Fox Foundation site, the Parkinson’s UK, the website of Toronto Western Hospital, then the movement disorders website.”*

In some cases, participants went beyond self-education to actively engage with the PD community. MP10(55yr) decided to join an advisory board and took the initiative to form their own support group.

***Lifestyle adjustments:*** Most participants mentioned that they had to make changes in their everyday activities and/or plans after PD diagnosis. These lifestyle changes provided insights into the ways participants adapted to the challenges of PD and highlighted the variability in individual responses to the disease.

Participants explained how their plans had to be canceled or limited due to the unexpected disease prognosis. For example, MP5(75yr) shared:

*“The projects that I had thought that I would do in my retirement would probably be limited or made impossible by Parkinson’s.”*

FP6(70yr) spoke about how PD made them lose interest in their favorite activities, she mentioned:

*“It affected it in the way that I just became apathetic to everything. I didn’t have any more desire to photograph.”*

#### The role of social relationships in a PD patient’s life.

Social relationships played a pivotal role in shaping participants’ responses to their PD diagnosis and their ongoing journey with the condition. This theme revealed the dual nature of social relationships as they could provide vital emotional and practical support but also contribute to psychological distress, especially when linked to painful personal histories with PD. This theme included two sub-themes: family and friends support and PD history of family and friends.

***Family and friends support:*** Participants were affected by social relationships including family and friends who provide various kinds of support. They expressed how fortunate they were because of the emotional support of their families which helped them manage the challenges of the disease.

MP2(69yr) shared how his grandchildren positively influenced his perspective:

*“I’m fortunate to have my grandchildren, so all of that really affected my outlook for my Parkinson’s, whatever comes I I’m not worried, just face life.”*

In some cases, the involvement of family members provided essential emotional encouragement. FP4(56yr) emphasized how her daughter’s regular check-ins reduced feelings of isolation, she shared:

*“My daughter checks in on me almost every day. Even if it’s just a short call, it really makes me feel like I’m not alone in this.”*

***PD history of family and friends:*** Participants with a family history of PD often recalled harsh memories of family members with the disease, which intensified psychological stress and anticipatory anxiety. Having a family member or a friend with a painful PD history could cause anticipatory stress as participants fear ending up with a similar disease prognosis. The following quote captured the moving words of FP1(54yr) who had a family history of PD:

*“I watched my father diagnosed young at the age of 42. He couldn’t move anything except his eyes, and I saw him deteriorate. So, when I heard Parkinson’s, of course, I went to that place, and I can’t even find words to describe it. Honestly, it was worse than a death sentence because it was like I was looking at increasing infirmity of a very severe, humiliating, unpreventable disease that there was nothing I could do about it.”*

Similarly, FP7(69yr) reflected on how her mother’s late-stage PD shaped her own fears about the future:

*“My mom had Parkinson’s and I remember her in the late stage. That image is always in my mind. I see it and it’s hard to shake. It makes me worry about what’s coming for me.”*

MP5(75yr) noted how having PD family history made him more anxious about potential changes in his own condition, he shared:

*“I keep thinking, is this the year I’ll stop being able to enjoy the things I love? What might happen to me, when I will end up being like him [his relative who had PD]?”*

#### The interaction of PD patients with the healthcare system.

The healthcare system played a critical role in shaping the experiences of participants living with PD. From the diagnosis process to ongoing care, interactions with the healthcare system profoundly influenced participants’ perspectives on their journey with PD. Each participant had unique circumstances that influenced their diagnosis story. This theme highlights two key sub-themes: waiting time and the role of healthcare providers.

***Waiting time:*** How long PD patients had to wait to receive medical services highly impacted their perspective on the healthcare system’s efficiency. Delays in receiving appointments and access to care added to participants’ stress and frustration, particularly during the critical period following diagnosis. MP9(70yr) expressed the challenges of limited access to his neurologist:

*“I only get to see my neurologist once a year, and in between, it’s just so hard to reach out if I’ve got any questions or worries.”*

Similarly, MP5(75yr) shared how a long wait time for a referral delayed his care:

*“When I was walking, my left arm wasn’t swinging very much. So, she [his family doctor] suggested that I go to the clinic, but that took 18 months to get an appointment.”*

Another participant said that they were able to avoid the long waiting time by accepting being enrolled in a drug trial.

***Role of healthcare providers:*** The roles of healthcare providers including general practitioners, neurologists, nurse practitioners, and physiotherapists, were pivotal in introducing participants to available support services. Participants described both positive and negative interactions which shaped their overall experience of the healthcare system.

Two participants mentioned how their healthcare providers referred them to a pilot support program which included a neurologist, a nurse, and a physiotherapist. MP10(55yr) expressed how useful this program was, he mentioned:

*“I really liked this program and I don’t regret joining it. It made me feel like I had a bit more control over my physical symptoms, and honestly, even my emotions.”*

Other participants mentioned that their neurologist gave them pamphlets and brochures about the available support services. Additionally, the role of a mental health nurse was highly influential for FP7(69yr), she said:

*“She [the mental health nurse] understood the anxiety and I had a few sessions with her which turned my world around to tell you the truth”*.

#### Support services available for recently diagnosed PD patients.

Support services played a vital role in alleviating the psychological stress experienced by recently diagnosed PD patients. This theme was focused on the support services available, identified barriers to their use, and highlighted participants’ suggestions for improvement. The theme is organized into three sub-themes: current support services, barriers to support services, and patient-suggested improvements.

***Current support services:*** Participants talked about the support services they used to help manage their psychological stress. Several participants mentioned joining Parkinson’s Canada support groups and some of them formed their own sub-groups. FP6(70yr) emphasized the importance of addressing specific needs:

*“I joined a women worldwide support group. It’s not through Parkinson’s Canada… You know, women have their own set of women’s issues and that’s why I joined one of these other groups.”*

Two participants enrolled in a 6-week pilot education program offered by Parkinson’s Canada which they found useful, especially in the early days after the diagnosis. Moreover, most of the participants highlighted the role of physiotherapy in decreasing their stress levels and addressing their PD emotional needs.

***Support services barriers:*** Participants discussed some of the barriers that limited their access to or willingness to engage with the current support services. FP4(56yr) shared a personal barrier, stating:

*“I certainly didn’t feel any desire to go to a support group or start talking to other Parkinson’s sufferers.”*

Health administrative barriers were also mentioned, with MP8(71yr) explaining:

*“You have to have your doctor fill out a form to get the disability tax credit. Lots of doctors don’t fill it out properly or don’t care and aren’t supportive. So, that’s a problem. That’s a barrier.”*

Access-related issues further compounded the challenges, as FP3(65yr) noted:

*“The barriers include how do you make some of these services readily available to the people who are two to three hours away. It’s not just these services are available but they’re financially available and they are physically available.”*

***Patient suggested improvements:*** Upon asking the participants about what was missing in the current support services that could have helped decrease their psychological stress, they suggested several improvements.

A recurring recommendation was strengthening communication channels with the neurologists, receiving regular updates on the latest PD research, and developing a centralized platform for PD information resources. MP12(52yr) suggested a structured program:

*“Launching a program called Parkinson’s 101 where they are not just having the information to read but having somebody there to interact with and talk to and explain it.”*

Participants also suggested a 24/7 PD hotline for patients to seek immediate support. where they can talk and be heard. Others suggested surveying patients about their preferences including age, disease stage, and gender to create more homogenous support groups.

## Discussion

This study aimed to provide insight into the psychological stress that recently diagnosed PD patients experience due to prognostic uncertainties and explore the gaps in the current support systems available for them and recommend areas of improvement based on the patients’ suggestions to address gaps in the support services. To reveal the details of the unique experiences of these patients, we conducted a qualitative study using in-depth interviews with 13 participants diagnosed with PD within 6 months to 5 years of the study start date. The participants shared their feelings, thoughts, and challenges during the early days of their PD journey. They were also eager to reflect on their emotional needs related to PD, discuss the causes behind these needs, and suggest system improvements that can benefit them and other PD patients.

The findings of this study align with Uncertainty in Illness Theory (UIT) which frames uncertainty as a critical determinant of psychological stress in chronic illnesses [28]. Participants’ experiences with prognostic uncertainties such as fears about disease progression, loss of autonomy, or becoming a burden, reflected UIT’s characterization of uncertainty as a stressor. Moreover, the varied coping strategies observed including information-seeking and engaging with support groups, corresponded to UIT’s adaptive processes [28,29]. This theoretical perspective underscored the importance of addressing uncertainty directly through tailored interventions such as improving patient education, fostering better communication with healthcare providers, and offering personalized support resources. By integrating UIT into the interpretation of our results, this study highlighted the value of reducing uncertainty as a means of alleviating psychological stress in recently diagnosed PD patients. Notably, addressing psychological stress is particularly important, as recent research suggests that stress-reducing interventions may have not only symptomatic benefits but potentially disease-modifying effects as well [44].

Most participants reported highly unfavorable experiences following their PD diagnosis, characterized by negative emotions and heightened psychological stress. These findings correspond with a previous study demonstrating a similar negative impact upon receiving a PD diagnosis which was described as “dropping the bomb” [45]. This psychological burden can also contribute to the disease’s well-established psychological difficulties including depression, anxiety, and sleep disturbances [9,44,46].

Although the main clinical spotlight is on the disease’s motor symptoms [9], participants revealed the harsh emotional consequences of receiving the PD diagnosis; participants stressed that they need to be equally addressed in recently diagnosed patients. Previous research has similarly noted that anxiety is often perceived by PD patients as more debilitating than motor symptoms, with the potential to exacerbate them [47]. Moreover, anxiety has been reported to limit patients’ lifestyles, underscoring its profound effect on overall well-being [47]. This also aligns with recent guidance from the British Psychological Society, which emphasizes the importance of early psychological interventions for individuals with neurodegenerative conditions, including Parkinson’s disease, to address emotional and cognitive challenges that often accompany the diagnosis [48].

Throughout our interviews, it was evident that this unpredictable nature of PD has a direct or indirect effect on the mental well-being of the affected individuals. We found that when patients first receive the diagnosis, different thoughts first cross their minds. Several patients expressed feelings of denial, sadness, and shock. However, one of the most common early stressors after diagnosis is the apprehension of becoming a liability to their families; one participant even considered medically assisted death to avoid that possibility altogether. As described by Hayes (2019), the unexpected nature of the disease can elevate the burden of declining mental health that usually accompanies PD [49].

One of the key findings was that the mental burden seemed to be fueled by prognostic uncertainties. Because of this bleak picture of the future, patients became concerned and afraid of what tomorrow may hold for them. This matches what Samii et al. described in the literature when stating that the disease uncertainties drive PD patients to become apprehensive and anxious about the future [16]. Furthermore, such uncertainties have been shown to exacerbate PD symptoms, including pain, fatigue, and functional disability, while also accelerating the development of adverse consequences [50]. The findings of this study and evidence in the literature suggest these unanswered questions contribute greatly to elevated stress levels in newly diagnosed patients.

Our findings show that despite overall similarities between patients, the experience with PD may follow different trajectories based on intrapersonal factors that shape each patient’s journey. Occupational factors and health literacy levels were particularly evident among the determinants of patients’ responses. Our results suggest educational levels may not be necessarily related to informed decision-making. For instance, a participant with a clinical background reported misdiagnosing himself, which further delayed receiving a formal diagnosis. Another participant who worked as a lawyer refused to seek help entirely. This is consistent with a previous report showing that knowledge gaps and misconceptions may be prevalent among PD patients irrespective of their education levels [51]. Similarly, another study identified barriers related to uncertainty in illness among PD patients, particularly those influenced by social and professional identities [20].

These examples highlight the complex impact of personal characteristics in shaping initial reactions to PD. However, we acknowledge that the small sample size limits our ability to draw broader conclusions about the influence of intrapersonal variables on the overall PD population. Our aim is to gain a comprehensive understanding of the specific experiences and challenges faced by the participants in this study. We acknowledge that these insights are representative only of these individuals and their unique contexts. While the findings offer valuable and detailed perspectives, they are intended to provide depth of understanding rather than broad generalizability.

Another key finding in our study was the divergent role of social relationships in PD patients’ lives. Several participants highlighted the positive impact of support from family and friends in managing their condition. However, for some, prior experiences with PD, particularly as caregivers for loved ones in advanced stages of the disease, introduced an additional layer of psychological stress. Caregiving for a PD patient is an intensely demanding role, often resulting in caregiver burden, which encompasses physical, emotional, mental, and socio-economic challenges [52]. Recalling the harsh memories and witnessing the progression of PD in their loved ones frequently evoked fears of reaching a similar stage and becoming a comparable burden themselves.

To the best of our knowledge, our study is the first to shed light on this aspect of psychological stress that PD patients may experience after diagnosis. Therefore, it is crucial to be conscious of the family history of recently diagnosed PD patients, which will allow for tailored psychological support and mitigate the risk of high psychological stress. This aligns with existing literature, which highlighted the pivotal role of social support as a key resilience factor [44]. Social support was shown to strengthen an individual’s capacity to manage the psychological stress associated with PD, offering both emotional and practical resources to effectively navigate the diverse challenges of PD [44].

The interaction of PD patients with different elements of the healthcare system is a key determinant of their experience with PD. Our study has explored various aspects of these interactions, including wait times and support resources. For instance, we found that long waiting times between neurologist appointments may contribute to psychological stress among recently diagnosed PD patients. This is consistent with data reported by Parkinson Canada, which found the average waiting time to see a Parkinson’s specialist to be 11 months, further contributing to the patients’ mental decline [53].

Additionally, previous literature emphasizes the critical role of the diagnostic journey in shaping patients’ psychological experiences [21]. For example, the social processes involved in early-onset PD diagnosis illustrate how diagnostic delays can intensify feelings of frustration and helplessness [21]. In our study, most participants described the period following their PD diagnosis as highly unfavorable, marked by negative emotions and heightened psychological stress. Another study highlighted the importance of delivering PD diagnoses with compassion and hope, which could influence patients’ psychological adjustment and coping mechanisms[22]. While resolving issues related to long waiting times may require systemic and large-scale changes in the healthcare system, improving the support offered by healthcare providers may be a faster and more viable strategy to reduce psychological stress in this population in the meantime.

Healthcare providers can address the gap between recently diagnosed PD patients and the available support services by educating patients about these services and referring them to other specialists, including physiotherapists and psychotherapists. Recent literature has increasingly emphasized the role of nonpharmacologic treatment strategies in reducing psychological stress among PD patients. Interventions such as physical exercise and mind-body practices have gained attention for their potential benefits [44,54]. We identified several support services commonly used by PD patients in Ontario, including support groups and a pilot educational program offered by Parkinson’s Canada. Interestingly, several participants formed their own support groups based on common interests, age, gender, or disease stage. Similarly, Phillips (2006) proposed creating support groups based on age and disease onset to improve these programs’ effectiveness [45]. Therapeutic group singing was also reported in the literature as an effective treatment modality for PD patients [55]. This approach successfully addressed physical, psychological, and social needs, fostering increased social interaction and support within a therapeutic and motivating environment [55].

In addition, we identified several barriers to using support services, including personal factors such as lack of the desire to join the support groups, and healthcare provider-related barriers and access-related issues. The Participants suggested several improvements to overcome these barriers, including fostering communications between patients and healthcare providers. Previous studies emphasized the importance of healthcare professionals providing clear and consistent medical information and employing effective communication strategies [20]. Such approaches were crucial in helping patients articulate their questions and anxieties while ensuring they receive detailed and reassuring responses from healthcare providers [20].

Participants also suggested developing tailored programs to meet their needs. For example, a participant proposed a program called “Parkinson’s 101” that integrates interactive education sessions with opportunities for patients and caregivers to ask questions and receive tailored advice. Such programs could enhance engagement and understanding while reducing psychological stress. To address systemic challenges, this could be implemented in partnership with existing organizations like Parkinson’s Canada and regional health networks, ensuring alignment with current resources and expertise.

Another suggestion was the establishment of a 24/7 hotline for immediate emotional and practical support for PD patients. Feasibility considerations for this solution could include leveraging telehealth infrastructure and training healthcare professionals or volunteers to provide both informational and emotional support. Partnerships with telemedicine providers or funding through public health initiatives could enhance its sustainability and scalability. Similarly, telehealth interventions were shown to improve cognitive functioning and positively impact the quality of life for both PD patients and their caregivers [56].

Participants suggested creating homogenous support groups based on factors like age, gender, and disease stage to foster a sense of relatability and shared experience. While this approach could address personal barriers to group engagement, it would require careful consideration of logistical challenges such as recruiting participants with similar characteristics and ensuring consistent attendance. Digital platforms such as virtual group meetings, could mitigate geographical barriers and expand access to such tailored groups. This suggestion aligns with the recommendations in the literature advocating for individualized psychological models that emphasized self-compassion as a resilience factor in managing chronic illnesses like PD [14,20]. Homogenous support groups could provide a safe and empathetic space where participants can share their experiences and challenges and foster a sense of mutual understanding and connection. Such environments could naturally foster self-compassion by alleviating feelings of isolation and stigma while promoting shared coping strategies. Individualized psychological interventions such as those using positive psychology, could be integrated into these groups to enhance their emotional benefits by building self-compassion and resilience while addressing shame and promoting self-kindness [50,57].

By exploring these gaps and recommended solutions, our study highlights the critical need to improve support services and ensure they are accessible, comprehensive, and tailored to the needs of PD patients.

## Clinical implications

The findings of this study highlight the critical need for incorporating psychological support into the routine care of recently diagnosed PD patients. Addressing the psychological stress caused by prognostic uncertainties should be a key component of early disease management. Integrating mental health resources such as access to counselors or tailored educational programs within PD care pathways may help alleviate emotional burdens and improve overall well-being. Moreover, enhancing communication channels between patients and healthcare providers could address common concerns and uncertainties, offering timely reassurance and decreasing stress. Developing holistic support programs that combine informational, emotional, and peer support could better cater to the diverse needs of PD patients. These programs should focus on providing personalized interventions to assist PD patients in navigating the psychological challenges of their diagnosis.

## Recommendations for future research

This study highlights the need for further research to expand the understanding and management of psychological stress in recently diagnosed PD patients. Future research should explore how psychological stress evolves over time and its impact on disease progression and quality of life. Additionally, investigating the effectiveness of specific interventions such as targeted counseling, digital health tools, or structured support groups, would provide valuable insights into improving mental health outcomes for this population. Lastly, examining the influence of family dynamics and caregiver support on mitigating PD psychological difficulties could offer new perspectives on holistic care approaches for PD patients.

## Limitations

Due to the COVID-19 social restrictions, there were challenges with conducting informative discussions and study interviews. Therefore, we conducted our initial informal discussions and interviews virtually through Microsoft Teams. However, this option had the potential to affect establishing rapport with the participants, which in turn could have affected their willingness to share their difficult situations and display their vulnerability.

We recruited a limited sample of participants from a single center (The movement disorder clinic at TWH). Patients from other clinics may have different resources available to them around the time of receiving PD diagnosis and they may have different experiences. Therefore, the collected data may not represent the needs of those patients from different areas with a different set of resources.

Moreover, the study aims at exploring potential approaches to decrease psychological stress for recently diagnosed patients. Due to the nature of the disease and current diagnostics, there is a potential gap between disease onset and diagnosis, especially with the lack of specific diagnostic tests for PD [58]. Therefore, recently diagnosed PD patients may be at various stages of disease progression leading to approaches that may not be equally effective for all the targeted patients. Additionally, the decision not to include treating physicians in the study, while intended to create a safe space for participants to discuss their experiences openly, may have had implications for establishing patient-clinician rapport and trust.

The relatively short recruitment period may have limited the diversity of the sample and restricted the range of experiences captured. Furthermore, potential recall bias, particularly among participants interviewed five years after their diagnosis, may have influenced the accuracy and detail of the shared experiences. The fading or alteration of memories over time could have impacted the reliability of the data regarding early stressors and challenges.

## Conclusion

PD patients experience psychological challenges, especially in the early years following diagnosis when prognostic uncertainties are at their peak. Our findings highlight the need for tailored support systems and inclusive patient education programs to address diverse needs while eliminating barriers to accessing existing services. A patient-centered approach is vital to ensure these support services effectively meet their intended goals.

## Supporting Information

S1 Appendix The interview guide.
(PDF)

S2 Appendix The coding framework.
(PDF)

S3 Appendix Consolidated criteria for reporting qualitative studies (COREQ): 32-item checklist.
(PDF)
