# Supplementary material for: Psychological stress associated with prognostic uncertainties in recently diagnosed Parkinson’s disease patients: A qualitative study
Source: PLoS One. 2025 Mar 11;20(3):e0319576. doi: 10.1371/journal.pone.0319576 (PMC11896053; doi:10.1371/journal.pone.0319576)
Supplement: S1 Appendix — (PDF) [file pone.0319576.s001.pdf]

S1 Appendix – Interview guide

Demographic data:

- Age:
- Gender:
- Date of diagnosis:

List of questions:

- **Tell me about your experience receiving the PD diagnosis.**
  Probes:
- – Could you please elaborate more on...?
- – What did you mean by…?
- – What was it like receiving the right diagnosis after being misdiagnosed with another disease? (Only if misdiagnosed)
- **How did it feel to learn the diagnosis of PD?**
  Probes:
  - – What was the main source of stress at this time?
  - – What was the effect of psychological stress on your everyday life activities?
  - – What are the changes you had to make in your life after the PD diagnosis?
- **What did you know about PD at the time when you received your diagnosis?**
  Probes:
  - – Tell me more about the effect of having a family member who has/had PD on your perception of being diagnosed with PD (If applicable)
  - – What are the resources you explored to learn more about PD?
  - – To what extent did you find exploring these resources useful to you?
- **What were the support services offered to you after you received the PD diagnosis?**
  Probes:
  - – How did you hear about these support services? Have you found any other support services?
  - – How long did it take to access these services?
  - – To what extent were these services useful in decreasing the psychological stress associated with the PD diagnosis?

- **In your opinion, what are the missing support services that could have helped you decrease the psychological stress associated with the PD diagnosis?**
  Probes:
  - Why do you think these services would help recently diagnosed PD patients?
  - How do you think these services can be integrated into the current system?
  - What are your expectations/suggestions for a potential PD support program for recently diagnosed PD patients?
