# Supplementary material for: Psychological stress associated with prognostic uncertainties in recently diagnosed Parkinson’s disease patients: A qualitative study
Source: PLoS One. 2025 Mar 11;20(3):e0319576. doi: 10.1371/journal.pone.0319576 (PMC11896053; doi:10.1371/journal.pone.0319576)
Supplement: S2 Appendix — (PDF) [file pone.0319576.s002.pdf]

S2 Appendix. The Coding Framework

Number	Code name	Description
1	First experience	Describing patients' initial feelings after receiving the PD diagnosis
2	Early Stressors	Includes all the psychological stressors that affected patients after receiving the PD diagnosis
3	Waiting time	Describing the times when patients were required to wait to receive PD primary care
4	Change in activities/plans	The changes that PD patients had to make in their everyday activities or future plans following the PD diagnosis
5	Self-support/educating	How patients learned about Parkinson's disease and found/initiated support services on their own
6	Prognostic uncertainties	The disease uncertainties associated with the disease progression - whether personal, professional, or social difficulties.
7	Diagnosis story	Describing how patients were diagnosed with PD
8	Support services barriers/facilitators	The barriers for patients to use support services – whether personal, accessibility issues, or healthcare provider barriers
9	Family/Friend PD history	Includes the impact of having a family member or a friend who was diagnosed with PD on recently diagnosed PD patients
10	Healthcare providers' role	Describing the role of healthcare providers including GP, neurologists, nurse practitioner, and physiotherapist in introducing patients to available support services
11	Patient's suggested improvements	Includes patients' suggestions on what is missing in the support services that could have helped decrease the psychological stress associated with the PD diagnosis
12	Family/friends support	Describing how patients' family and/or friends can help support them in their PD journey
13	Personality/background types	How personality and/or background impact PD patient's experience including personality type, health literacy level, and current/past profession
14	Self-reflection	People reflecting on learning something about themselves
15	Current support services	Includes the support services offered by the Canadian PD associations that patients are using to help them on their PD journey
